# Supplementary material for: Integrating Mental Health Management into Empowerment Group Sessions for Out-of-School Adolescents in Kenyan Informal Settlements: A Process Paper
Source: Int J Environ Res Public Health. 2024 Feb 14;21(2):223. doi: 10.3390/ijerph21020223 (PMC10888709; doi:10.3390/ijerph21020223)
Supplement: Supplementary file 1 [file ijerph-21-00223-s001.zip › ijerph-2535605-supplementary.pdf]

Supplementary S1: FORMATIVE RESEARCH GUIDE

Bridging the Gaps: Mental Health Support for Out of School Adolescents in Urban Slums in Kenya

<u>IDI Guide for Adolescents</u>

Warm Up

Thank you for taking time to talk to me today. We're going to spend some time talking about mental health especially in adolescents including what mental ill-health means to you, its impact on peoples' lives, what current services are there in the community to help those suffering from mental ill-health and what additional programs do you think would improve the lives of those suffering from mental health especially your peers and youth as suchi. It should take around 1 hour. First, can we start off by telling me a bit about where you come from, how long you have lived in [name of neighborhood] and your favorite thing about living in this community?

Now let's talk about mental health…

Personal knowledge and perceptions of mental health and illness:

- What does "mental health" mean to you?
- What do you think are the causes of mental ill-health, especially in adolescents?
- How do you feel adolescents suffering from mental illness or ill-health should be treated?
- What are some beliefs and attitudes that are held in your community about mental health and people suffering from mental health?
- Do young people develop mental illnesses as a reaction to social and familial problems? Could you talk about these?

Personal experiences with mental health illness

- Do you personally know anyone suffering from mental ill-health, including yourself?
- How are you/they treated by your/their family and friends?
- How are you/they treated by the community?
- What type of mental health support/ services do you/ they have access to within the community you/ they live in (both medical and non-medical)?
- How do peers support each other? What role can young people play in supporting one another?

If no to the above (does not know anyone with mental health and does not suffer from mental health)….

Imagine you had a mental health illness:

- How do you think you would be treated by your family and friends?
- How do you think you would be treated by members of the community?
- What type of mental health support do you think you will have access to within the community you live in (both medical and non-medical)?

For everyone…

- How do you feel the COVID-19 pandemic has impacted your mental health?
- Are these suggestions for interventions that can help you feel better supported in your behavioral and mental health?

Community based interventions to support adolescents suffering from mental health

- What changes if any, would you like to see in the way mental health and illness are viewed <u>in this community</u>, especially in young people? What do you think would bring about these changes?
- What changes if any, would you like to see in access to mental health services (both medical and non-medical) <u>in this community</u>, especially for young people? What would bring about these changes?
- How can family members and the community be more supportive of those who are suffering from mental ill-health?

Wrap Up

We have come to the end of the interview. Before we finish, is there anything else that you would like to add about what we have discussed?

- Do you have any other questions that you'd like us to answer?

Thank the respondent for their time. Explain that the information they provided will be used to improve the implementation of the BRIDGING THE GAPS program. Explain to them that should there be need to clarify any information or get additional information from them, we will contact them for another interview.

Supplementary S2: FORMATIVE RESEARCH FINDINGS DISCUSSION

Before commencing the study, a formative research was carried out aiming at qualitatively understanding the knowledge, attitudes, and access to care of out-of-school adolescents with mental illness residing in urban informal settlements. 42 virtual in-depth interviews were conducted with out-of-school adolescent participants, their parents, and mentors from each site. The interviews were recorded with participant consent and transcribed then coded by two independent qualitative analysts using Atlas.ti software into themes and sub-themes. A select number of the transcripts were also double-coded to ensure common understanding and interpretation and an acceptable inter-coder agreement was also reached for reliability (coefficient of .70).

We learnt that several terminologies were used to describe mental illness. For example, most adolescents, parents and mentors thought it a state of depression. Other adolescents described mental illness as a state of insanity for example using words such as 'madness' with fewer respondents describing it as a state of being stressed. We noticed that the mentors associated it with stress and defined it as a health condition affecting the mind and behavior due to stress. When asked what they perceived as the causes of mental illness in adolescents, they gave three major sub-themes of social, financial and family as categories.

When asked about the type of mental health support or services that adolescents suffering from mental illness had access to within the community and how peers with such challenges support one another, they reported that parents and caregivers had inadequate information on how to manage and support adolescents with mental illness. In terms of accessibility of professional services, respondents reported limited knowledge about mental health facilities within the community. They were unaware of the available community health volunteers offering supportive services to adolescents with mental illness. However, some enrolled in rehabilitation centers, and few received treatment and medication from hospitals.

Suggestions for improving access in the existing health care facilities in the community included improving capacity to manage mental illness by providing mental health services in the health facilities. Establishing counselling and rehabilitation centers was cited with most respondents mentioning the need to ensure that they were free or affordable and youth friendly. The scaling up and establishment of an information hub and call center for mental health issues within community was also recommended.

Aside from health facilities, participants reported the need to educate parents/caregivers on home management of mentally ill adolescents, conducting community awareness on mental illness events to empower them to support families with need was suggested. Sensitization of parents and adolescents on how to detect mental illness in their adolescents and seeking early treatment were recommended.

Other suggestions included peers within the community regularly discussing issues that could contribute to mental illness amongst themselves and referring those among them to relevant facilities for professional care. Forming support groups for parents and caregivers of adolescents with mental illness and providing social support as well as education on how to deal with emerging issues were included.

Providing material support for such adolescents to ensure that they were comfortable, identifying and nurturing their potential, empowering them with technical skills for generation of income, engaging them in community

projects, and eradication of drug use within the community which precipitates mental illness were other suggestions given.
